# Enhanced Magneto-Optic Properties in Sputtered Bi- Containing Ferrite Garnet Thin Films Fabricated Using Oxygen Plasma Treatment and Metal Oxide Protective Layers

**DOI:** 10.3390/ma13225113

**Published:** 2020-11-12

**Authors:** V. A. Kotov, M. Nur-E-Alam, M. Vasiliev, K. Alameh, D. E. Balabanov, V. I. Burkov

**Affiliations:** 1 Institute of Radio Engineering and Electronics, Russian Academy of Sciences, 11 Mohovaya St, Moscow 125009, Russia; kotov.slava@gmail.com; 2 Electron Science Research Institute, Edith Cowan University, Joondalup, WA 6027, Australia; vasiliev.mikhail@gmail.com (M.V.); k.alameh@ecu.edu.au (K.A.); 3 Moscow Institute of Physics and Technology, 9 Institutski Per., Dolgoprudny 141700, Russia; dima-mipt@mail.ru (D.E.B.); optikcentr@mail.mipt.ru (V.I.B.)

**Keywords:** magneto-optics, mcd, faraday rotation, figure of merit, polarization, oxygen plasma treatment

## Abstract

Magneto-optic (MO) imaging and sensing are at present the most developed practical applications of thin-film MO garnet materials. However, in order to improve sensitivity for a range of established and forward-looking applications, the technology and component-related advances are still necessary. These improvements are expected to originate from new material system development. We propose a set of technological modifications for the RF-magnetron sputtering deposition and crystallization annealing of magneto-optic bismuth-substituted iron-garnet films and investigate the improved material properties. Results show that standard crystallization annealing for the as-deposited ultrathin (sputtered 10 nm thick, amorphous phase) films resulted in more than a factor of two loss in the magneto-optical activity of the films in the visible spectral region, compared to the liquid-phase grown epitaxial films. Results also show that an additional 10 nm-thick metal-oxide (Bi_2_O_3_) protective layer above the amorphous film results in ~2.7 times increase in the magneto-optical quality of crystallized iron-garnet films. On the other hand, the effects of post-deposition oxygen (O_2_) plasma treatment on the magneto-optical (MO) properties of Bismuth substituted iron garnet thin film materials are investigated. Results show that in the visible part of the electromagnetic spectrum (at 532 nm), the O_2_ treated (up to 3 min) garnet films retain higher specific Faraday rotation and figures of merit compared to non-treated garnet films.

## 1. Introduction

Since several decades ago, magneto-optic (MO) applications of garnet materials were well-known. Bismuth (Bi)-substituted garnet materials for various MO applications attract the attention of researchers in this field, aimed at developing innovative high-performance garnet materials or finding ways of improving their properties. Also, from the practical point of view, MO garnet materials of these composition types with high-performance are relevant to the context of manufacturing of next-generation ultra-fast optoelectronic devices, such as light intensity switches and modulators, high-speed flat panel displays and high-sensitivity sensors [1,2,3,4,5,6,7,8,9,10,11,12,13,14]. Therefore, it is important, nowadays, to obtain MO materials of optimized material composition stoichiometry possessing a high figure of merit and a low coercive field. This can typically be achieved with garnets having high Bi-substitution levels. In recent years, significant research activities have been reported in the field of the manufacture and characterization of new-generation ultrathin films of yttrium iron garnet (YIG) and related materials, of thicknesses ranging from several nanometers to several tens of nanometers. The record-low optical losses of YIGs in the ultrahigh frequency (UHF) spectral region make them attractive for the development of spintronics and modern microwave devices. The low losses of YIGs are due to the small damping parameter α ≈ 3 × 10^−5^ (the ferromagnetic resonance linewidth of less than 0.5 Oe at 9 GHz). Thin films of ferromagnetic metals lag behind YIG materials in performance (in terms of the damping parameter characteristics) by two orders of magnitude, thus enabling a significant reduction in switching current for spin valve devices, which are the key components used to develop magnetic sensors, hard disk read heads and magnetic random access memories (MRAM) [1]. Another potential practical area that can benefit from the use of thin YIG films is related to the development of different nano-electronic device types, which utilize the phenomenon of spin current generation by magnetostatic spin waves propagating in thin YIG films possessing small damping parameters [5]. Bismuth-substituted iron garnets, being ferrimagnetic dielectrics possessing giant specific Faraday rotation across the visible and near-infrared spectral regions, also represent the most promising MO materials for use in different MO devices, such as magnetic photonic crystals (MPC), non-reciprocal MO elements, Faraday-effect ultrafast MO modulators, magnetic field-controlled multilayer MO waveguiding structures, hybrid multiferroics-based multilayers and other applications in photonics [15,16,17]. From the point of view of the practical applications of ferrite garnets in hybrid integrated-optics circuits, the most promising fabrication approach is RF magnetron sputtering of amorphous-phase garnet films onto substrates such as gadolinium gallium garnet (GGG), kept at room temperature (or between 100–400 °C), followed by annealing crystallization processes run at temperatures between 490–650 °C. The properties of a transitional layer forming between the substrate and deposited film are a defining factor, which governs the annealing crystallization process since the crystallization processes of these garnet film structures start from the transitional layer region. When using the thin or ultrathin ferrite garnet layers in bilayer-type structures involving garnet film as spin wave generator and a nanoscale platinum film as spin current detector, at the forefront is the problem of the uniformity of the magnetic properties in thin or ultrathin garnet films and whether these also possess record-low damping parameters or small ferromagnetic resonance (FMR) linewidths near 1 Oe. In addition to this, the application of additional or protective layers or films is very useful for the topographic mapping of the sensitive media [18,19,20]. There are literature reports, presenting the data showing that in thin and ultrathin iron-garnet films, there exist significant variations in both the composition and magnetic properties across the film thickness [5]. For example, near the substrate-film boundary region, in epitaxially-grown iron garnet films, dependent on growth conditions, complex transitional layers of thickness ranging between several nm up to 250 nm, may form [21]. It is important to note that in these films, the transitional-layer thickness (as evaluated using Curie temperature measurements), may also reach 250 nm. With increasing film thickness, up to 2 µm, a constant Curie temperature value is observed. 

On the other hand, oxygen plasma treatment is an attractive and widely used technique on both the experimental and industrial scales to improve thin-film technology without introducing any complexity into the material stoichiometry [22,23,24]. Oxygen plasma treatment is a well-known method used to clean the substrates for the development of thin films, since the oxygen plasma treatment enhances the adhesion of thin films to the substrates. The optimized oxygen plasma treatment process can enhance the thin film’s bonding strength (surface energy) and adequately modify the film surfaces for various microelectronics and optoelectronics devices without affecting the entire nanostructure of the devices [22,23,24,25,26,27]. J. W. Roh et al. have reported that the oxygen plasma- assisted wafer bonding process is very effective and crucial for the fabrication of integrated optical waveguide isolators. They treated the surfaces of GGG substrates by oxygen plasma for 30 s with a radio frequency (RF) plasma power of 100 W under oxygen pressure of 0.3 Torr and observed high bonding strength and hydrophilicity without any voids in the interface in bonding of Indium phosphide (InP) thin films to Gd_3_Ga_5_O_12_ (GGG) substrates [26]. K. H. Chen et al. have applied the oxygen plasma treatment process in low temperature environment to improve the electrical and physical properties of as-deposited (Ba_0.7_Sr_0.3_)(Ti_0.9_Zr_0.1_)O_3_ (BSTZ) thin films [27]. They have reported that the oxygen plasma treatment affects the chemical bonding state and crystalline structure to help reduce the density of interface states, oxygen vacancies and defects for as-deposited BSTZ thin films and enhance the capacitance of the films. Growing high-quality thin films of various oxide and metal-oxide-based materials, including MO garnets, on various substrates, is typically accomplished by creating oxygen plasma and allowing extra oxygen input with argon (Ar), Nitrogen (N_2_) or Hydrogen (H) or Helium (He) plasma during the deposition process [28,29,30,31,32,33,34,35,36,37]. However, to the best of our knowledge, using post-deposition oxygen plasma treatment on as-deposited highly Bi-substituted iron garnet thin films, prior to the annealing crystallization processes, has never been reported, at least not in conjunction with MO quality measurements.

To improve the properties of highly Bi-substituted metal doped iron garnet thin film materials, we propose two new and modified process sequences for annealing crystallization of garnet thin films. The new method for the manufacture of high-performance ultrathin garnet films is the provision of a thin (2–20 nm) protective bismuth oxide (Bi_2_O_3_) layer, which assists in the crystallization of the garnet layer. In this case, during the initial stage, a 20–60 nm amorphous-phase film of a nanocomposite co-sputtered material type of (Bi_2_Dy_1_Fe_4_Ga_1_O_12_ + Bi_2_O_3_), is deposited onto a GGG or a glass substrate. The excess bismuth oxide content relative to the stoichiometric composition of ferrite garnet is kept between 10–40 vol %. The second stage involves the deposition (also by RF sputtering) of a protective bismuth-oxide layer of thickness between 2 nm–20 nm onto these amorphous nanocomposite films. The obtained two-layer structure is then subjected to annealing crystallization in an air atmosphere, at a temperature between 490 °C and 650 °C, for 1 h. Results show that the MO performance characteristics of the samples (nanocomposite material of composition type Bi_2_Dy_1_Fe_4_Ga_1_O_12_ + Bi_2_O_3_ with a protective Bi_2_O_3_ layer) exceed, by more than a factor of two, the corresponding parameters obtained in identical material systems fabricated without this additional protective layer. We also report on the studies of the Faraday rotation and its dispersion (conducted in the 400 nm–600 nm interval), as well as the magnetic circular dichroism (MCD, performed in between 300 nm–600 nm). Results demonstrate a two-fold improvement in the MO characteristics of oxide-protected garnet films, due to both the increased bismuth substitution levels and the prevention of bismuth evaporation from the subsurface film regions. 

Secondly, we apply oxygen plasma treatment on as-deposited garnet samples immediately after deposition and then follow the previously established (composition-dependent) high-temperature annealing processes to crystallize the garnet thin films. We investigate the effects of post-deposition oxygen plasma treatment on the MO properties of RF sputtered garnet thin-film layers, synthesized using two different types of Bi-substituted garnets. The oxygen plasma treated and non-treated garnet thin films are characterized and analyzed after running the annealing processes. In the conducted experiments, we repeatedly noticed that the post-deposition low- temperature oxygen plasma treatment improves their material properties, especially the Faraday rotation per unit film thickness and the optical absorption coefficients, thus leading to obtaining a higher MO figure of merit compared to that of non-treated annealed garnet layers.

## 2. Background and Transitional Layer Properties

When using a single solution-melt, depending on the epitaxial growth temperature and the supercooling magnitude, the transitional layer thickness can vary widely, from 5 nm to around 250 nm, with the transitional layer being possibly composed of several intermediate layers. For example, a growth regime with bismuth-containing solution-melt supercooling near ΔT ~ 150 °C at a growth temperature around 750 °C leads to the appearance of an intermediate transitional sublayer of thickness around 100 nm at the substrate-film boundary (Curie temperature of the iron garnet composition being 225 °C). As a result, in this thickness interval, the epitaxial growth process occurs under the conditions that the growth rate is being limited by the crystallization rate at the substrate-film boundary. Past this stage, a thick transitional sublayer appears, of thickness near 150 nm, within which the Curie temperature reduces from 225 °C to 215 °C. At the same time, for this material, the effective field of the uniaxial magnetic anisotropy, defined as H_k_^eff^ = H_k_ − 4πM_s_, changes smoothly from H_k_^eff^ = 1500 Oe at the epitaxial layer thickness h = 30 nm, to H_k_^eff^ = 2100 Oe, at h = 250 nm. Studies of the lattice parameter dependency on the epitaxial layer thickness conducted in the thickness range between 250 nm and 1µm for Bi-substituted ferrite garnet films showed that both the Curie temperature of material and the lattice parameter do not change and are equal to T_C_ = 215 °C and a_f_ = 12.401 Å, respectively. Within the starting region of the transitional layer, the corresponding measured values were T_C_ = 225 °C and a_f_ = 12.412 Å, respectively [1]. According to the data reported in Reference [5], an increase in the Bi substitution by 1 formula unit (f.u.) within epitaxial films has led to an increase in the garnet lattice parameter by Δa_f_ = 0.0828 Å. Therefore, increasing the Bi-substitution from 0.3 f.u. to 1.43 f.u. should lead to an increase of the transverse lattice parameter from a_f_ = 12.401 Å to a_f_ = 12.412 Å and hence, the data on the T_c_ and lattice parameter near the film-substrate boundary do not match well [1,38]. 

At a growth temperature around 980 °C and solution melt supercooling of around ΔT = 5 °C, it is possible to grow an epitaxial garnet layer of thickness around several microns, within which the Curie temperature remains practically constant across the entire volume of the epitaxial layer. It is important to note that, when fabricating thin and ultrathin bismuth-substituted iron-garnet layers, the formation of transitional layers near the film-substrate boundary may also take place due to the partial amorphization of the substrate surface occurring during the pre-deposition argon-plasma bombardment as a result of additional substrate-cleaning measures. The Ar^+^ ion energies may, in this case, reach between tens of eV to several keV. Another cause of the significant changes in the composition of film with thickness and the related changes in the magnetic properties of films, is the annealing crystallization procedure, which takes place within the (composition-dependent) temperature range from 490 °C to 650 °C [1]. Etching of GGG substrates undertaken prior to the epitaxial film growth leads, at best, to the root mean squre RMS surface roughness of the substrate surface being near ~0.25 nm. Usually, in Liquid Phase Epitaxy (LPE)-grown iron garnet films fabricated while keeping constant melt temperature during growth, a significant reduction of the Bi substitution content is observed across the film layer thickness, towards the direction of the film-air boundary. During the experiments aimed at finding the optimum temperature of epitaxial growth, it has been found that, at a growth temperature between 950 °C and 980 °C and melt supercooling near ΔT = 5–10 °C, it is possible to manufacture films with a constant Curie temperature, within ΔT_c_ = 3 °C [1]. In this study, several batches of Bi-containing thin-ferrite garnet-type films are fabricated and characterized in order to better understand the annealing crystallization processes for the synthesis Bi-substituted ferrite garnets (which initially are found to be in an amorphous phase after RF magnetron deposition). 

## 3. Garnet Layers Sputter-Deposition and Annealing Process and Parameters 

Multiple batches of single-layer bismuth-substituted garnet compounds doped with dysprosium and gallium and bi-layer structures (garnet layer covered by a top thin protective oxide layer) have been prepared on glass and monocrystalline garnet substrates using the RF magnetron sputtering technique. The sputtering targets used had nominal compositions of Bi_2_Dy_1_Fe_4_Ga_1_O_12_, Bi_2.1_Dy_0.9_Fe_3.9_Ga_1.1_O_12,_ Bi_1.8 L_u_1.2_Fe_3.6_Ga_1.4_O_12 and_ Bi_2_O_3_. From our previous work, we had found that the films of co-sputtered composition type (Bi_2_Dy_1_Fe_4_Ga_1_O_12_ sputtered with excess Bi_2_O_3_) possessed simultaneously a high Faraday rotation and the necessary level of uniaxial magnetic anisotropy to orient the magnetization of the films in the direction perpendicular to the film plane [5]. 

Some of the as-deposited garnet layers were treated with oxygen plasma exposure immediately after the deposition process before the high temperature crystallization process was performed. The process parameters used to prepare the garnet layers, including sputter deposition, oxygen plasma exposure and annealing crystallization, are detailed in Table 1. O_2_ plasma treatment was conducted using YZD08-5C plasma cleaner (purchased through Alibaba.com) for 0.5–5 min. The plasma-treated and the non-treated samples were then annealed by using the optimized annealing regimes found in previous annealing experiments for this composition of garnet layers [3,4]. The film quality and the properties of annealed garnet samples were first characterized in terms of the specific Faraday rotation and MO figure of merit at 532 nm. 

In this work, the post-deposition annealing processes were run using a conventional temperature-controlled and heating-rate-controlled oven in the temperature range of 490 to 650 °C.

Several batches of simple (double and triple)-layer-type all-garnet heterostructures were also manufactured, investigated and their optimized process parameters and properties (i.e., optimizing the heterostructure annealing regimes and characterizing the crystallization behavior, inter-material compatibility and microstructural properties) were reported by our group. Our previously published data confirmed the annealing crystallization behavior of Bi-substituted iron garnet and garnet-oxide composites deposited onto various substrate types [3,4,5]. 

## 4. Results

### 4.1. Spectral Dependencies of Faraday Rotation Measured for Nanocrystalline Films of Composition Bi_2_Dy_1_Fe_4_Ga_1_O_12_

**Figure 1:**
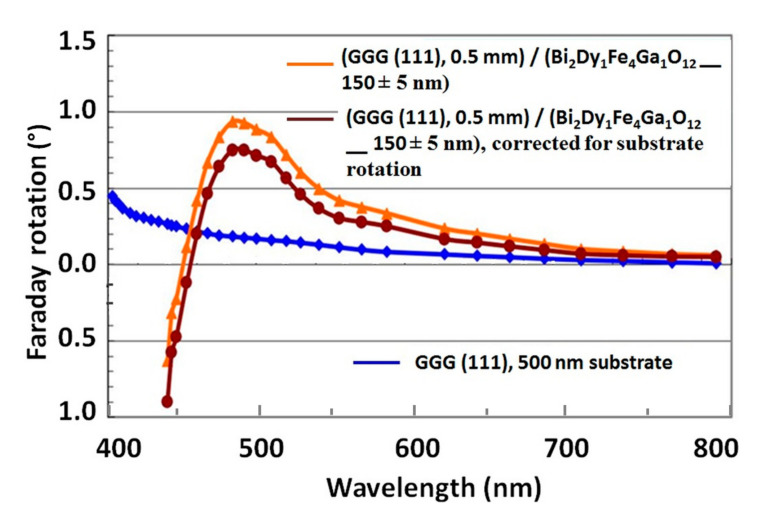
Spectral dependencies of Faraday rotation measured for nanocrystalline films of composition Bi_2_Dy_1_Fe_4_Ga_1_O_12_ at the saturated magnetization state.

Figure 1 shows the spectral dependency of the specific Faraday rotation, where a peak at 494 nm is observed, for a Bi_2_Dy_1_Fe_4_Ga_1_O_12_ film of around 150 nm thickness. When measuring the MO characteristics in samples of less than 20 nm thickness, across the temperature interval between 8K–200K (−265.15 to −73.15 °C), we observed magnetic circular dichroism (MCD) spectra similar to these typical for nanocrystalline Bi-substituted ferrite garnets with thicknesses between 500–1000 nm. During this study, we measured the MCD spectra between 250–600 nm. 

Y. Sun et al. has reported PLD-manufactured YIG films on GGG substrates with FMR linewidth of 3.4 Oe, defined as the interval between the extrema of the derivative of the FMR absorption line at 10 GHz. The film surface roughness determined by scanning probe microscopy was between 1–3 nm [39]. The years 2010 and 2011 witnessed the birth of a new paradigm in the discipline of spintronics—“spintronics using yttrium iron garnets [1,15,16,17,40,41]. The significance of this research field originates from two features of yttrium iron garnet (Y_3_Fe_5_O_12_, YIG) materials: (1) extremely small damping factor and (2) electrically-insulating property. 

### 4.2. Scpectral Dependencies of Magnetic Circular Dichroism for Standard Nanocomposite-Type Samples of Bi_2_Dy_1_Fe_4_Ga_1_O_12_ + Bi_2_O_3_

**Figure 2:**
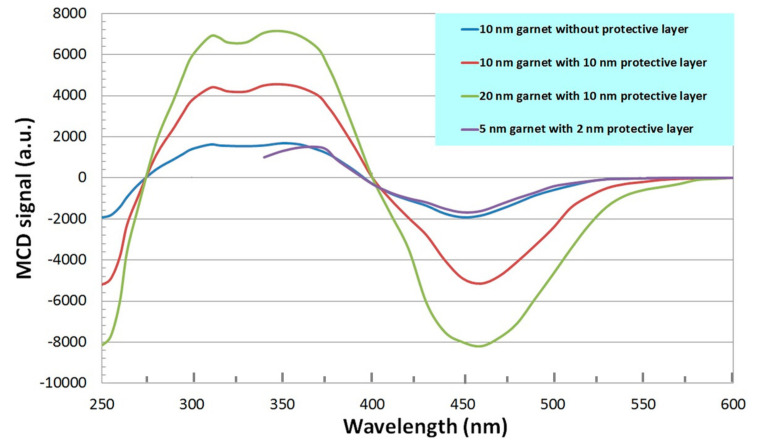
Spectral dependences of the magnetic circular dichroism of ferrite garnet films, in the spectral range from 250 to 600 nm.

In this study, we measured the magnetic circular dichroism (MCD) spectra in the wavelength range of 250–600 nm in nanocomposite films of bismuth-containing ferrite garnets with an excess of bismuth oxide. The results of studying the spectral dependence of magnetic circular dichroism in a nanocomposite film of the Bi_2_Dy_1_Fe_4_Ga_1_O_12_ + Bi_2_O_3_ system in the spectral range from 250 to 600 nm are shown in Figure 2. The sign of the MCD effect is opposite to the sign of MCD observed in films of ferrite garnets of composition (YBi)_3_Fe_5_O_12_ [40]. This is because the studied sample has a magnetic compensation point at a temperature above room temperature. Note that, for (YBi)_3_Fe_5_O_12_ samples, the tetrahedral magnetic sublattice of a ferrite garnet is oriented along the applied magnetic field, however, by the substituting yttrium ions by dysprosium ions and iron ions by gallium ions in the dodecahedral ferrite garnet sublattice reverse the magnetization orientation.

### 4.3. Bi_2.1_Dy_0.9_Fe_3.9_Ga_1.1_O_12_ and Bi_1.8_Lu_1.2_Fe_3.6_Al_1.4_O_12_ Garnet Layers Annealed Followed by Post-Deposition Oxygen Plasma Treatment

**Figure 3:**
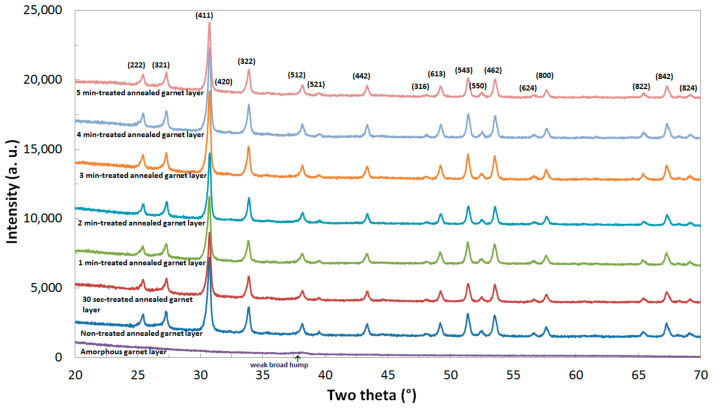
Measured X-ray diffraction (XRD) patterns in as-deposited and annealed Bi_2.1_Dy_0.9_Fe_3.9_Ga_1.1_O_12_ garnet layers.

X-ray diffraction (XRD) traces obtained for as-deposited and annealed Bi_2.1_Dy_0.9_Fe_3.9_Ga_1.1_O_12_ films using a Siemens 5000D x-ray diffractometer are shown in Figure 3. The presence of a weak broad hump at ∼38.2° in the XRD pattern of the as-deposited garnet samples is attributed to the amorphous phase of the samples just after deposition as well as to the post oxygen plasma exposure. However, following annealing at 580 °C and higher, the broadened hump at 38.2° is turned into a small but significantly noticeable, peak (512) together with a number of stronger peaks consistent with the primary garnet phase representing their crystallization stage and the nanocrystalline microstructure of the garnet films. All identified XRD peaks and their angular positions with the half maximum-line width (FWHM) values were determined using the Jade 9 (MDI Corp.) software package (Peak-listing option). The lattice constant and crystallite sizes for the synthesized garnet-type materials were calculated using the standard procedures followed in Ref [3]. It can be concluded that all the garnet layers present the crystalline phage however, from our experiments we observed that the Oxygen plasma treated samples were able to annealed at a comparatively low temperature compared to that of the non-treated garnet samples.

**Figure 4:**
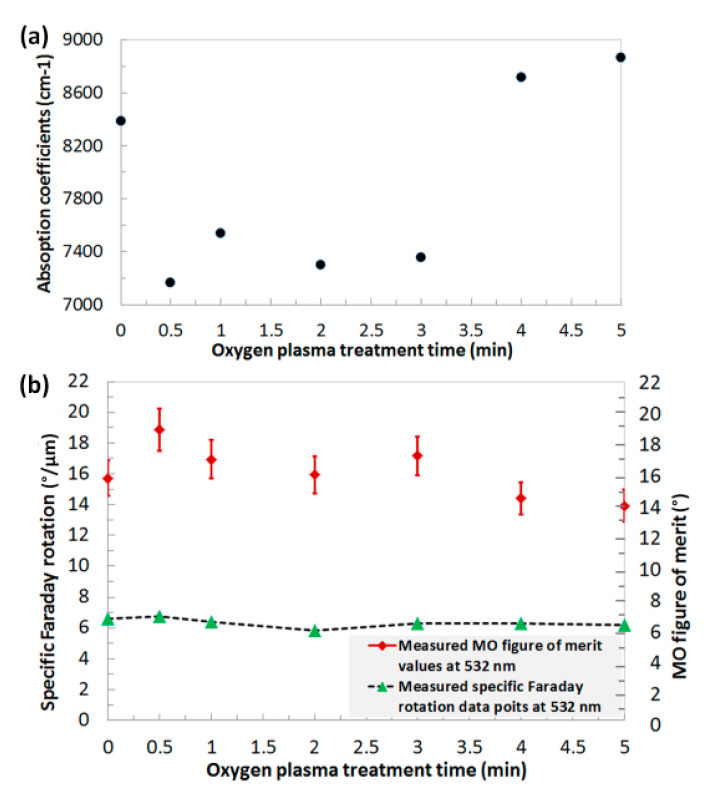
Measured optical absorption coefficients (**a**), specific Faraday rotation and magneto-optic (MO) figure of merit data points (**b**) at 532 nm in optimally annealed O_2_ plasma treated and non-treated Bi_2.1_Dy_0.9_Fe_3.9_Ga_1.1_O_12_ garnet layers.

**Figure 5:**
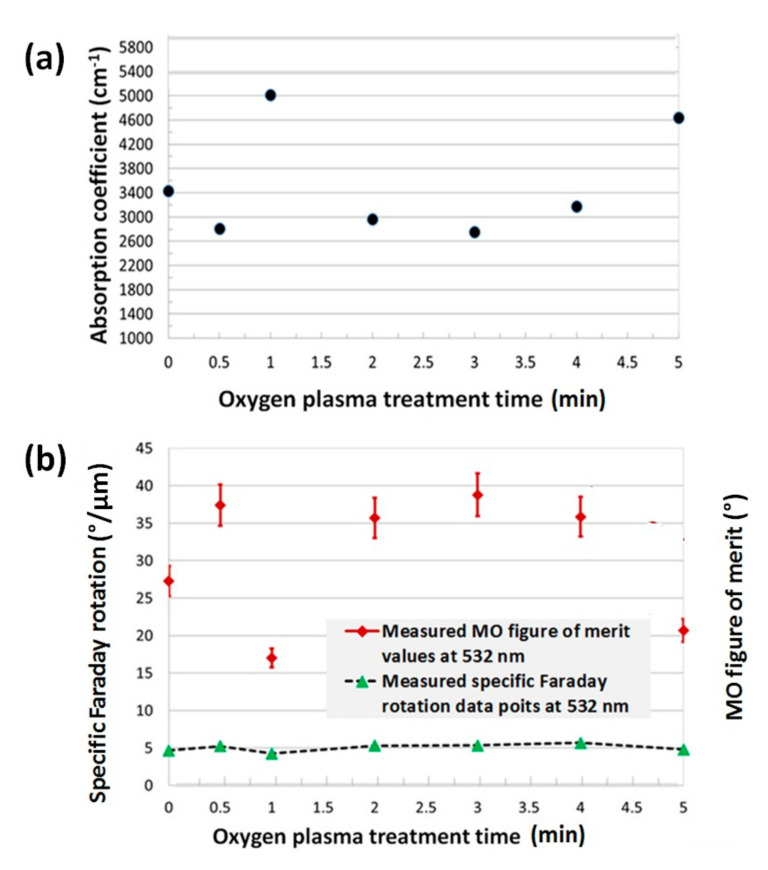
Measured optical absorption coefficients (**a**), specific Faraday rotation and MO figure of merit data points (**b**) at 532 nm in optimally annealed O_2_ plasma treated and non-treated Bi_1.8_Lu_1.2_Fe_3.6_Al_1.4_O_12_ garnet layers.

To investigate the effects of post-deposition oxygen plasma treatment on the optical and magneto-optical behaviours of Bi_2.1_Dy_0.9_Fe_3.9_Ga_1.1_O_12_ type thin films, we derived the optical absorption coefficients, measured the specific Faraday rotation and calculated the MO figure of merit at a certain spectral wavelength (532 nm). The transmission spectra of all annealed garnet layers were spectrally fitted with the modeled transmission spectra to determine the optical absorption on the garnet layers by using the MPC software reported in Reference [42]. The plasma-treated samples showed slightly higher specific Faraday rotation at 532 nm compared to the non-treated garnet samples and this was repeatedly observed in all batches of plasma-treated annealed samples. However, significantly lower optical losses (optical absorption) were observed in the O_2_-plasma-treated (up to 3 min) garnet layers than that of non-treated garnet films, thus leading to an improved MO figure of merit ($Q=2\times \Theta_{F}/\alpha \;$, where $\Theta_{F}$ is the specific Faraday rotation and α is the absorption coefficients) as shown in Figure 4 and Figure 5. Estimated errors in films’ thicknesses (within ± 5% accuracy) as well as in Faraday rotation were accounted for during the calculations of the MO figures of merit. 

The best MO properties were obtained in the sample that was exposed to oxygen plasma for only 30 s inside the high vacuum chamber. This sample showed the highest specific Faraday rotation, lowest optical absorption at 532 nm. The highest MO figure of merit, about 19 degrees, was obtained in the 30-s plasma-treated garnet layer of composition type Bi_2.1_Dy_0.9_Fe_3.9_Ga_1.1_O_12_ whilst the highest MO figure of merit, above 35 degrees, was obtained in plasma-treated garnet layers of composition type Bi_1.8_Lu_1.2_Fe_3.6_Al_1.4_O_12_. It is important to note that the specific Faraday rotation slightly decreased with increasing the plasma treatment time, however, the absorption coefficient increased significantly with the plasma exposure time, and, consequently, reduced the MO quality of the garnet layers after 3 min. 

**Figure 6:**
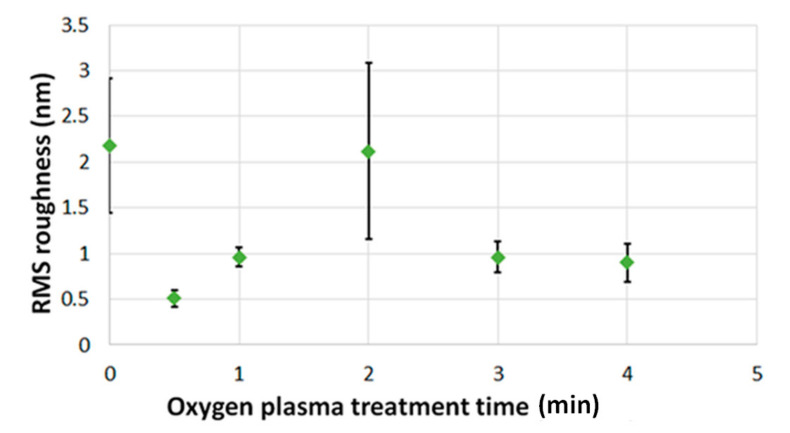
Measured root mean square RMS surface roughness values vs. oxygen plasma exposure time, for the various developed garnet thin films.

The surface roughness profile of the garnet thin films (oxygen plasma treated and non-treated) were studied and it was found that all annealed samples exhibited smooth, uniform and consistent morphology across film surfaces except some minor micro-cracks (occurred due to substrates high-temperature expansion and also for the lattice mismatch of the garnet layer and the substrate). Garnet films exposed to oxygen plasma for 30 s possessed the best surface quality with RMS roughness value (Rq) of around 0.5 nm, as can be seen in Figure 6. This indicates that such films have significant surface effects while other samples including the non-treated garnet layer showed RMS value over 1 nm. From the above discussion. it can be concluded that the O_2_ plasma treatment effectively interacts with the material surface layer and reduces the surface roughness without intruding into the entire layer structure. This changes the surface energy of the garnet layers and helps the garnet layers get more oxygen diffusion during the annealing crystallization process, leading to better MO properties compared to the non-treated garnet layers. Note that, films treated with O_2_ plasma for longer than 30 s displayed surface larger nanoscale grain features. 

It was observed that the MO figure of merit decreased with the increase in plasma treatment time though the specific Faraday rotation remained higher compared to that of the non-treated annealed garnet layer. From the overall experimental datasets, we can conclude that the oxygen plasma treatment helps the amorphous garnet layers to crystallize with less effect added from foreign contaminants on the layer surface, compared to the non-treated samples. This is an ongoing research work and will be continued in order to develop new garnet materials with improved MO properties for existing and emerging applications in magneto photonic, magneto-plasmonic and integrated optics.

## 5. Conclusions

Studies of the magneto-optical properties of ultrathin RF magnetron sputtered bismuth-substituted iron garnet films have been conducted in the temperature interval from room temperature down to 8K (−265.15 °C). For the first time, the effects of thin protective Bi_2_O_3_ layers on the MO properties of ultrathin highly bismuth-substituted dysprosium iron garnet layers have been investigated. At room temperature and also at cryogenic temperatures, the spectral dependencies of magnetic circular dichroism have been measured between 250–850 nm. At room temperature, the MCD spectra typical of Bi-substituted ferrite garnets have been measured (in samples without protective oxide layers), in films of thickness 10.3 nm and above. 

In order to improve the MO quality in ultrathin nanocomposite films of co-sputtered composition type Bi_2_Dy_1_Fe_4_Ga_1_O_12_ + Bi_2_O_3_, we have introduced a new modification of the annealing crystallization process, in which a 10 nm-thick protective layer of Bi_2_O_3_ served to prevent the films from Bi content loss otherwise occurring during the high-temperature annealing. Results show that the magnitude of MCD signal measured at 450 nm an oxide-protected annealed film of system Bi_2_Dy_1_Fe_4_Ga_1_O_12_ + Bi_2_O_3_, is 2.7 times higher than that of a Bi_2_Dy_1_Fe_4_Ga_1_O_12_ unprotected by an oxide layer. The properties of the garnet thin films can also be improved by employing the oxygen plasma treatment just after the deposition process. Additionally, we believe that pre-diffusing the oxygen from plasma into garnet films volume prior to annealing could lead to better (faster) compensation of oxygen loss occurring during sputtering, thus preventing the excessive formation of non-garnet material phases during annealing. However, this preliminary finding may require further experimental confirmation.

## Figures and Tables

**Table 1:** Summary of process parameters used to prepare the garnet layers.

Sample Preparation Stage	Process Parameters	Values & Comments
Garnet layers deposition	Sputtering target stoichiometry oxide-mixed garnet targets	Bi_2_Dy_1_Fe_4_Ga_1_O_12_, Bi_2.1_Dy_0.9_Fe_3.9_Ga_1.1_O_12,_ Bi_1.8_ Lu_1.2_Fe_3.6_Ga_1.4_O_12__and_ Bi_2_O_3_
	Base pressure	4–5 × 10^-6^ Torr
	Argon (Ar) pressure	≈2 mTorr
	Substrate stage temperature	Room Temperature 21–23 °C
	Substrate stage rotation rate	16–17 rpm
Oxygen plasma treatment	Base pressure	750 mTorr
	Oxygen flow	0.2 sccm/min
	RF power densities	40 W
	Plasma exposure time	30 s to 5 min
